# Microarray analysis of microrna expression in peripheral blood mononuclear cells of patients with polymyositis and dermatomyositis

**DOI:** 10.2478/jtim-2022-0055

**Published:** 2024-05-21

**Authors:** Jia Shi, Shuang Zhou, Jiuliang Zhao, Dong Xu, Hui Huang, Mengtao Li, Xinping Tian, Linrong He, Chanyuan Wu, Qian Wang, Yan Zhao, Xiaofeng Zeng

**Affiliations:** Department of Rheumatology and Clinical Immunology, Chinese Academy of Medical Sciences & Peking Union Medical College; National Clinical Research Center for Dermatologic and Immunologic Diseases (NCRC-DID), Ministry of Science & Technology; State Key Laboratory of Complex Severe and Rare Diseases, Peking Union Medical College Hospital (PUMCH); Key Laboratory of Rheumatology and Clinical Immunology, Ministry of Education, Beijing 100730, China; Department of Respiratory Medicine, Peking Union Medical College Hospital, Chinese Academy of Medical Sciences & Peking Union Medical College, Beijing 100730, China; China-Japan Friendship Hospital, Yinghua East Road, Chaoyang District, Beijing 100029, China

**Keywords:** microRNA, dermatomyositis, polymyositis, interstitial lung disease

## Abstract

**Background and Objectives:**

MicroRNAs (miRNAs) represent a new class of biomarkers in the context of connective tissue disorders. The miRNA expression profiles in peripheral blood mononuclear cells (PBMCs) of patients with polymyositis (PM) and dermatomyositis (DM) have not been fully elucidated. The objective is to investigate miRNAs expression profile in PBMCs of patients with PM/DM.

**Methods:**

Microarray technology was used to identify differentially expressed miRNAs in PBMCs obtained from 6 untreated PM/DM patients and 3 healthy controls (HCs). TaqMan-based stem-loop real-time PCR detection was used for validation in a cohort of 34 PM/DM patients and 20 HCs.

**Results:**

Microarray analysis revealed 38 differentially expressed miRNAs (24 up-regulated and 14 down-regulated) in PM/DM patients compared to HCs. Four miRNAs (miR-320a, miR-335-3p, miR-34a-5p and miR-454-3p) were chosen for real-time PCR validation. The expression of miR-34a-5p was significantly upregulated in PM/DM group (*P* < 0.05). In subgroup analysis, miR-34a-5p was significantly upregulated in interstitial lung disease (ILD) group and DM group (*P* < 0.001). The level of SIRT1, a validated target of miR-34a, was significantly lower in PBMCs of PM/DM patients compared with HCs.

**Conclusions:**

MiR-34a-5p may potentially participate in the pathogenesis of PM/DM through SIRT1, and may serve as a potential new biomarker for PM/DM-ILD.

## Introduction

Idiopathic inflammatory myopathy (IIM) is a heterogeneous group of acquired autoimmune diseases affecting the skeletal muscle and many other organs, such as skin, lungs, and heart. Dermatomyositis (DM) and polymyositis (PM) are the most common subtypes of IIMs.^[1, 2]^ Early and accurate diagnosis of IIM is of much clinical significance for effective treatment and to prevent further episodes and complications such as interstitial lung disease (ILD). Muscle biopsy, which is routinely used for diagnosis of IIM, is an invasive test. It would be helpful to identify a less invasive auxiliary diagnosis. Moreover, the etiology and pathogenetic mechanisms underlying IIMs are not well characterized. It is generally accepted that IIMs occur as a result of exposure to environmental factors in genetically susceptible individuals.^[1]^ Identification of novel biomarkers may help in the management of patients and help unravel the pathogenesis of IIM.

MicroRNAs (miRNAs) are a class of small non-coding RNAs (average length: 22 nucleotides) which usually inhibit the translation of target mRNA by binding to the 3’ untranslated region (3’UTR) of the target.^[3]^ MiRNAs play important regulatory roles in various biological processes, including myogenesis and immunity.^[4]^ Dysregulated miRNAs have been reported in many autoimmune diseases, including systemic lupus erythematosus and rheumatoid arthritis, and this dysregulation may be linked to disease activity and pathogenesis.^[5, 6, 7]^ Studies have identified altered expressions of some miRNAs in IIM, such as decreased expression of miR-1, miR-133a, miR-133b,^[8]^ miR-7,^[9]^ miR-146a^[10]^ and miR-223^[11]^ in muscle or skin tissues, decreased expression of miR-23a-3p in serum,^[12]^ decreased expression of miR-4442 in plasma,^[13]^ and increased expression of miR-96-5p in whole blood samples.^[14]^ In terms of function, the downregulation of miR-21^[15]^, miR-381^[16]^, and miR-146a^[17, 18]^ have been reported to regulate macrophage migration in PM/DM. These altered expression profiles are associated with specific autoantibodies, clinical phenotypes and disease activity.^[12, 19, 20]^ The level of miRNA expression can be different in different tissue samples; *e.g*., miR-1 was reported to be upregulated in IIM serum,^[21]^ but downregulated in muscle tissues.^[8]^ In addition, there is a paucity of research on the miRNA profile of peripheral blood mononuclear cells (PBMCs) in patients with IIMs. In addition, whether miRNA profile of PBMCs is abnormal, remains to be elucidated.

In this study, we explored differentially expressed miRNAs in PBMCs isolated from PM/DM patients by microarray analysis and PCR validation, with a special interest in the association with ILD.

## Materials and methods

### Patients and control individuals

This study was approved by the Peking Union Medical College Hospital Ethics Committee. From July 2014 to April 2015, 40 patients with PM/DM and 23 healthy controls (HCs) were enrolled in this study. Patients and HCs were matched by sex and age. The diagnosis of PM/ DM was based on Bohan and Peter criteria.^[22]^ ILD was diagnosed according to the criteria of 2013 American Thoracic Society/European Respiratory Society.^[23]^ Informed consent has been obtained from all individuals included in this study.

### Blood sample collection, RNA isolation, and quality control

Peripheral blood samples were collected in EDTA-treated tubes, and PBMCs were isolated by standard Ficoll density gradient centrifugation. Total RNA was prepared using Trizol reagent (Invitrogen) following the manufacturer’s protocol. RNA yields and purity were determined by nanodrop spectrophotometer (ND-1000, Nanodrop Technologies) and RNA Integrity was determined by gel electrophoresis.

### RNA labeling and array hybridization

The isolated RNA was labeled with miRCURY™ Hy3™/ Hy5™ Power labeling kit (Exiqon, Vedbaek, Denmark), and was then hybridized on the miRCURY^TM^ LNA Array (v.18.0) (Exiqon). Each slide was scanned using the Axon GenePix 4000B microarray scanner (Axon Instruments, Foster City, CA, USA). Each miRNA spot was replicated for four times on the same slide.

### Array data analysis

Scanned images were then imported into GenePix Pro 6.0 software (Axon Instruments, Redwood City, CA, USA) for grid alignment and data extraction. Expression data were normalized using the median normalization. Significantly differentially-expressed miRNAs were identified through Volcano Plot filtering. Hierarchical clustering was performed using R Script.

### Reverse transcription and quantitative real-time PCR

8 ng total RNA was used per 12 μL reverse transcription reaction to produce cDNA using the TaqMan® MicroRNA Reverse Transcription Kit (Applied Biosystems, Foster City, CA, USA). MiRNA expression was quantified using TaqMan® microRNA assay primers for human miR-34a-5p, miR-320a, miR-335-3p, miR454-3p and U6 with TaqMan® Universal PCR master mix (Applied Biosystems). Fold changes for miR-34a-5p, miR-320a, miR-335-3p, and miR454-3p were calculated after normalization using U6 as endogenous control.

qRT-PCR of mRNAs was performed using ABI PRISM 7900HT (Applied Biosystems, Foster City, CA, USA) and SYBR® Premix Ex Taq™ II (Takara, Osaka, Japan). A total of 1 μg RNA from each sample was used to generate cDNA with the PrimeScript RT reagent kit (Takara, Osaka,Japan). Primer pairs used for real-time PCR are shown in Table 1. The results of qRT-PCR were normalized to β-actin expression.

**Table 1:** qRT-PCR primers used in the study

**Gene**	**Sequence**
SIRT1	5’-GATTGGCACAGATCCTCGAA-3’(forward)
	5’-GTCTACAGCAAGGCGAGCATA-3’(reverse)
β-ACTIN	5’-CATGTACGTTGCTATCCAGGC-3’(forward)
	5’-CTCCTTAATGTCACGCACGAT-3’(reverse)

### MiRNA target prediction and enrichment analysis

Three algorithms were used for miRNA target prediction, including miRanda, TargetScan 6.2, and mirBase. Only miRNA target genes identified by at least two of these algorithms were considered. DAVID was used to analyze the enriched Kyoto Encyclopedia of Genes and Genomes (KEGG) pathways using default settings.

### Statistical analysis

Normally distributed variables were presented as mean ± standard deviation (SD) and between-group differences assessed using *t* test. Non-normally distributed variables were presented as median (interquartile range) and between-group differences assessed using Mann-Whitney test. Two tailed *P* values < 0.05 were considered indicative of statistical significance. All statistical analyses were performed using SPSS 19.0 software (IBM Corp., Armonk, NY, USA).

## Results

### Profiling of miRNAs in PBMC by microarrays

**Figure 1:**
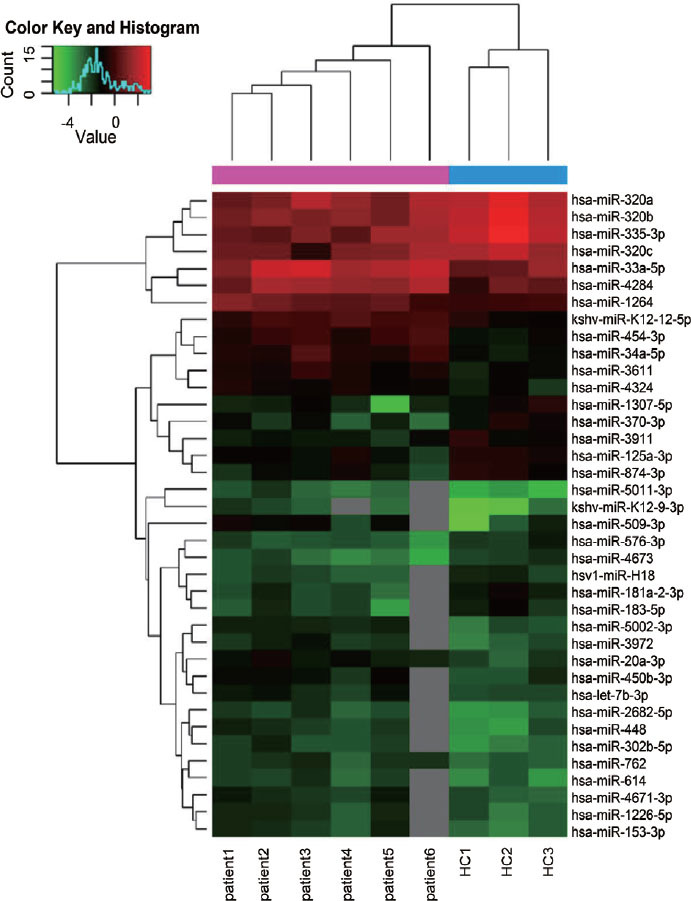
Hierarchical clustering analysis of the miRNA profiles of the PM/DM group (patient1, patient2, patient3, patient4, patient5, patient6) and control group (HC1, HC2, HC3). Downregulated miRNAs are presented in green, and upregulated miRNAs are presented in red. HCs: healthy controls; PM: polymyositis; DM: dermatomyositis.

We performed microarray analysis of PBMCs from 6 untreated PM/DM patients and 3 HCs. Compared with HCs, a total of 38 differentially expressed miRNAs were identified in PM/DM patients (fold change > 1.5, *P* < 0.05; Figure 1), with 24 upregulated and 14 downregulated (Table 2).

**Table 2:** Differentially expressed miRNAs in PBMCs from PM/DM patients compared with healthy controls

Differential expression type	miRNA name	Ratio of exp/con
Upregulated	kshv-miR-K12-12-5p	1.80
	hsa-miR-5002-3p	1.94
	kshv-miR-K12-9-3p	2.96
	hsa-miR-448	2.04
	hsa-let-7b-3p	1.60
	hsa-miR-509-3p	2.48
	hsa-miR-302b-5p	2.01
	hsa-miR-450b-3p	1.74
	hsa-miR-3972	1.89
	hsa-miR-3611	1.56
	hsa-miR-33a-5p	1.97
	hsa-miR-2682-5p	1.92
	hsa-miR-5011-3p	2.51
	hsa-miR-20a-3p	1.87
	hsa-miR-614	1.80
	hsa-miR-1264	1.72
	hsa-miR-1226-5p	1.64
	hsa-miR-4671-3p	1.69
	hsa-miR-153-3p	1.92
	hsa-miR-4284	2.12
	hsa-miR-4324	1.55
	hsa-miR-454-3p	2.07
	hsa-miR-34a-5p	2.09
	hsa-miR-762	1.57

Downregulated	hsa-miR-181a-2-3p	0.53
	hsa-miR-335-3p	0.37
	hsa-miR-1307-5p	0.54
	hsa-miR-320b	0.46
	hsa-miR-183-5p	0.55
	hsv1-miR-H18	0.66
	hsa-miR-874-3p	0.59
	hsa-miR-320c	0.53
	hsa-miR-370-3p	0.54
	hsa-miR-576-3p	0.61
	hsa-miR-125a-3p	0.67
	hsa-miR-320a	0.56
	hsa-miR-3911	0.62
	hsa-miR-4673	0.56

Ration of exp/con: the miRNA expression ratio of polymyositis and dermatomyositis patients compared with healthy controls.

### Validation of the miRNA expression by qRT-PCR

**Figure 2:**
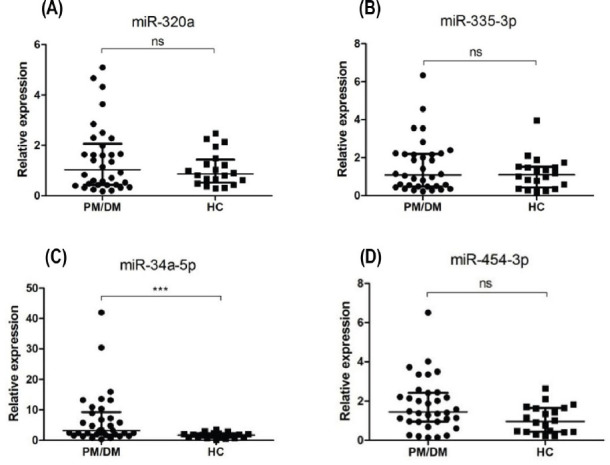
Differential expression of 4 miRNAs in the peripheral blood mononuclear cells from PM/DM patients (*n* = 34) and healthy controls (*n* = 20). (A-D) qRT-PCR results for miR-320a, miR-335-3p, miR-34a-5p, miR454-3p levels, respectively. Mean miR-34a-5p level in PM/DM patients was significantly higher than that in HCs. The relative expression levels were normalized to the expression of U6. *** *P* < 0.005; ns: no significance. HCs: healthy controls; PM: polymyositis; DM: dermatomyositis.

According to fold change and abundance, four miRNAs (miR-34a-5p, miR-320a, miR-335-3p, miR454-3p) were selected for stem-loop real-time PCR validation. The general characteristics of the validation population are presented in Table 3. The qRT-PCR results are shown in Figure 2. There was no significant difference between PM/DM patients and HCs with respect to the expression of miR-320a or miR-335-3p (1.03 [0.43, 2.06] *vs*. 1.06 ± 0.67, *P* > 0.05; 1.09 [0.47, 2.19] *vs*. 1.18 ± 0.87, *P* > 0.05; respectively). PM/DM patients had higher expression levels of miR-34a-5p and miR454-3p, which was consistent with microarray results. Mean miR-34a-5p level in PM/DM patients was significantly higher than that in HCs (3.20 [1.87, 9.24] *vs*. 1.68 ± 0.91, *P* = 0.001), but there was no significance between-group difference with respect to miR-454-3p level (1.44 [0.95, 2.42] *vs*. 1.06 ± 0.70, *P* = 0.053).

**Table 3:** Characteristics of the validation population

	PM/DM	HC
Number (N)	34	20
Age (mean ± SD, years)	42.5 ± 14.0	43.4 ± 10.8
Female (N)	22	13
DM (N)	27	-
ILD (N)	19	-

PM: polymyositis; DM: dermatomyositis; HCs: healthy controls; ILD: interstitial lung disease.

**Figure 3:**
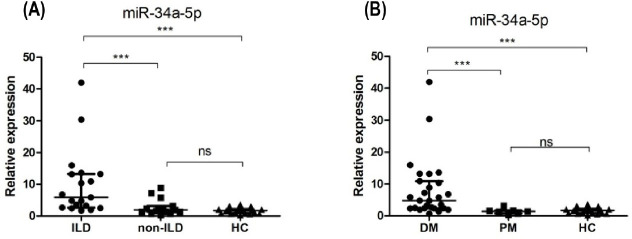
Clinical relevance of miR-34a-5p. (A) miR-34a-5p was significantly upregulated in the ILD group compared with non-ILD group or HCs. (B) miR-34a-5p was significantly higher in DM group compared with PM group or HCs. ***P < 0.005; ns: no significance. HCs: healthy controls; PM: polymyositis; DM: dermatomyositis.

Next, we explored the clinical relavance of miR-34a in ILD by further dividing the 34 PM/DM patients into ILD (*n* = 19; 5 male, 14 female; mean age 46 ± 11 years) and non-ILD subgroups (*n* = 15; 7 male, 8 female; mean age 37 ± 15 years). The expression level of miR-34a-5p was compared among the 2 subgroups and HCs (Figure 3). The miR-34a-5p was significantly upregulated in the ILD group compared with non-ILD group or HCs (5.85 [2.66, 13.22] *vs*. 1.94 [1.36, 3.13] *vs*. 1.06 ± 0.67); however, there was no significant difference between non-ILD group and HCs in this respect (*P* = 0.216). In addition, we also assessed the difference between DM subgroup (*n* = 27; 8 male, 19 female; mean age 44 ± 12 years) and PM subgroup (*n* = 7; 4 male, 3 female; mean age 35 ± 17 years). The level of miR-34a in DM group was significantly higher compared with PM group or HCs (4.81 [2.39, 10.92] *vs*. 1.42 [0.72, 1.68] *vs*. 1.06 ± 0.67); however, there was no significant difference between PM group and HCs in this respect (*P* = 0.569).

### MiRNA target prediction and qRT-PCR validation

**Figure 4:**
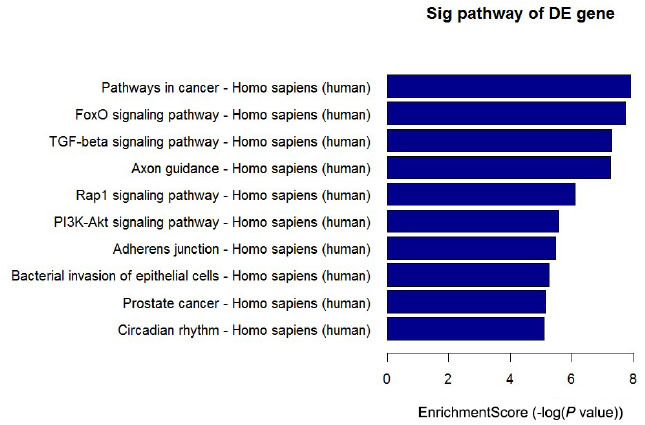
Top 10 significantly enriched pathways of target genes ranked by *P* value. The target genes of differentially expressed miRNAs were predicted by at least two out of three algorithms. KEGG pathway analysis was based on these genes.

MiRNAs can influence gene expression by causing translational repression or mRNA degradation, which can dysregulate the downstream pathways.^[4]^ Therefore, in this study, we performed miRNA gene target predictions using miRanda, Targetscan, and miRbase. A total of 11,564 targets were predicted, and based on these targets, KEGG pathway analysis was performed. The most enriched pathways included FoxO signaling pathway, TGF-beta signaling pathway, Rap1 signaling pathway, bacterial invasion of epithelial cells, PI3K-Akt signaling pathway, and pathways in cancer (Figure 4). This analysis can help understand the potential functions of the differentially expressed miRNAs.

**Figure 5:**
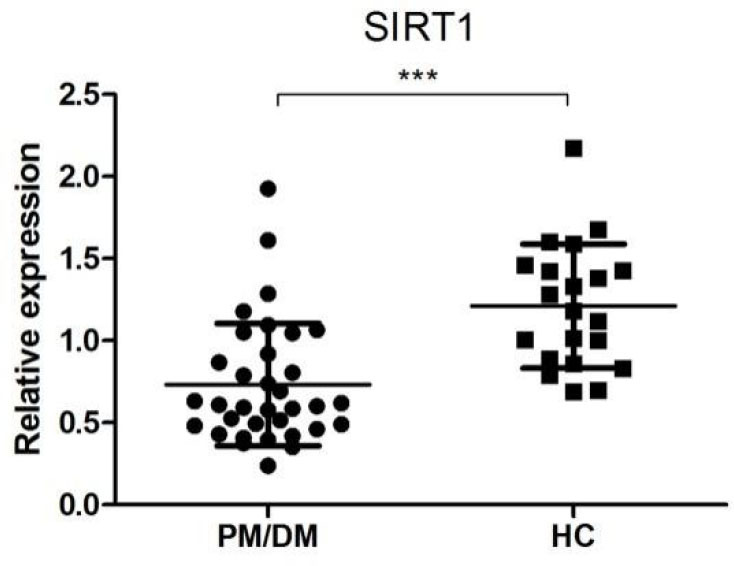
Relative expression of SIRT1 in PM/DM patients (*n* = 34) and healthy controls (*n* = 20). The relative expression levels were normalized to the expression of β-actin. ***P < 0.005. PM: polymyositis; DM: dermatomyositis.

SIRT1 is a validated target of miR-34a^[24]^ and is also a key molecule of the FoxO pathway,^[25]^ one of the most enriched pathways of dysregulated miRNAs’ targets. SIRT1 mRNA level in the PBMCs of PM/DM patients and HCs was then determined by qRT-PCR (Figure 5). The expression level of SIRT1 was significantly lower in PBMCs from PM/ DM patients than that from HCs (0.73 ± 0.37 *vs*. 1.23 ± 0.38, *P* < 0.001), which was in accordance with the negative regulatory role of overexpressed miR-34a.

## Discussion

Previous studies have identified differentially expressed miRNAs in the muscle and skin tissues of PM/DM patients.^[26, 27]^ However, the miRNA expression profile in PBMCs has not been fully elucidated. In this study, we investigated the overexpressed or suppressed miRNAs in PBMCs from PM/DM patients and identified 38 differentially expressed miRNAs compared with HCs by microarray analysis. PCR study further validated the overexpression of miR-34a in PBMCs from PM/DM patients.

ILD is one of the most common extra-muscular manifestations of PM/DM and is associated with poor prognosis.^[28, 29]^ Thus, identification of novel biomarkers, such as miRNA, which can facilitate early identification and risk-stratification of PM/DM-ILD, may help improve the outcomes for these patients. On subgroup analysis, we identified significant upregulation of miR-34a in the ILD group compared with HCs and non-ILD group, but there was no significant difference between non-ILD group and HCs. Besides, the level of miR-34a was significantly higher in DM group compared to that in PM group or healthy controls. Due to the higher frequency of lung involvement in the DM group (18/27) compared with PM group (1/7), it is not clear as to which factor predominantly determined the miR-34a level. Future studies should include a large sample of PM-ILD patients to provide more robust evidence of the relationship between miR-34a and PM/ DM-ILD.

MiR-34a is located in chromosome 1p36 and plays an important role in the p53 pathway.^[30]^ The protein deacetylase SIRT1 is a critical regulator of replicative senescence, inflammation, aging and immune responses.^[31, 32, 33]^ MiR-34a acts upstream of SIRT1 axis, and thereby inhibits translation of SIRT1 and eventually leads to cell apoptosis.^[24, 34]^ SIRT1 was shown to regulate inflammation by interaction with nuclear factor kappa-B (NF-ϰB). The activated form of SIRT1 directly inhibits NF-ϰB transcription *via* deacetylation of the p65 subunit of the NF-ϰB complex,^[33]^ and in turn, NF-ϰB downregulates SIRT1 deacetylase activity *via* expression of miR-34.^[35]^ The role of miR-34a has been reported in many autoimmune diseases. In multiple sclerosis, miR-34a was shown to be overexpressed in peripheral blood CD4+T lymphocytes, which resulted in decrease in suppressor of cytokine signaling 3 (SOCS3), leading to an increased production of cytokines and increase in Th17 cells.^[36]^ Overexpression of miR-34a was also shown to play an imperative role in rheumatoid arthritis, by attenuating *Foxp3* (phenotype marker of Treg cell) gene expression at the transcriptional level through targeting its 3’ UTR in CD4+ T cells.^[36, 37]^ Besides, abnormal miR-34a expression has been reported in ILD. In the lungs of patients with pulmonary fibrosis (PF), miR-34a expression was significantly higher in the type II alveolar epithelial cells (AEC), and this may dysregulate cell senescence^[38]^ and promote epithelial-mesenchymal transition.^[39]^ In addition to overexpressed miR-34a, increased p53 acetylation and decreased SIRT1 have also been reported in AECs in PF. Suppressing miR-34a in AECs was shown to inhibit bleomycin-induced p53 and prevent PF, whereas upregulating miR-34a increased p53 and apoptosis in AECs of mice unexposed to bleomycin.^[40]^ In our study, we also observed significantly decreased expression of SIRT1 mRNA in the PBMCs from PM/DM group compared with HCs. Therefore, upregulation of miR-34a-5p in the PBMCs may be a potential biomarker of PM/DM-ILD. These findings suggest the plausibility of the involvement of the miR-34a/SIRT1 axis in the pathogenesis of PM/DM-ILD.

Several limitations of our study should be considered while interpreting the findings. PM/DM is a heterogeneous disease with a variety of myositis-specific autoantibodies (MSAs). Patients with different MSAs may exhibit distinct miRNA expression profiles. Currently there is a trend for further grouping of PM/DM on the basis of MSAs. However, the facility for detecting MSAs was not available at our center at the time of collection of specimens for this study. Lastly, we did not assess imaging data for further classification of ILD.

In summary, our study identified differentially expressed miRNAs in the PBMCs of PM/DM patients, which may serve as novel biomarkers for PM/DM and provide new insights into the pathogenesis of IIM. Insights from our analysis may help further unravel the mystery of ILD in PM/DM.
